# Impact of Digital Navigation on Screening Adherence With the Multi-Target Stool DNA Test

**DOI:** 10.36469/001c.133939

**Published:** 2025-05-05

**Authors:** Mallik Greene, Timo Pew, A. Burak Ozbay, John B. Kisiel, A. Mark Fendrick, Paul Limburg

**Affiliations:** 1 Exact Sciences Corporation, Madison, Wisconsin, USA; 2 Exact Sciences, Madison, Wisconsin, USA; 3 Division of Gastroenterology and Hepatology Mayo Clinic, Rochester, Minnesota, USA; 4 Department of Internal Medicine University of Michigan, Ann Arbor, USA

**Keywords:** colorectal cancer, colorectal cancer screening, digital outreach, adherence, compliance, multi-target stool DNA test, mt-sDNA test

## Abstract

**Background:** Colorectal cancer (CRC) is the fourth most frequently diagnosed cancer and the second leading cause of cancer-related deaths in the United States. Screening can prevent CRC by detecting advanced precancerous lesions. Adherence to screening is crucial in reducing CRC disease burden; however, there is limited research on the impact of digital outreach screening uptake and adherence. **Objective:** This study evaluated the impact of different digital outreach channels on patient adherence to CRC screening with a multi-target stool DNA (mt-sDNA) test in a real-world setting. Methods: Patients were individuals aged 45 to 85 years with a valid mt-sDNA test order from Exact Sciences Laboratories, LLC (Jan. 1, 2023–Sept. 23, 2023). All patients received letters and phone calls; some received short message service (SMS), email, or both. Adherence and time to test return were compared across digital outreach categories stratified by patient characteristics. Multivariable regression evaluated the association of digital outreach methods with adherence and time to test return. **Results:** Among 2 425 308 patients (43.5% between 50 and 64 years, 58.2% female), digital SMS only (62.7%) was the most common outreach method. Overall adherence was 70.1%, with highest adherence in the digital SMS-plus-email group (72.9%). Mean time to test return from shipment of mt-sDNA kit to receipt of valid test was 25.8 days. In adjusted analyses, patients receiving digital SMS plus email had the highest odds of test return (odds ratio, 1.75; 95% confidence interval [CI], 1.73-1.78; *P*<.001) and had return times 8.7% shorter than the no-digital-outreach group (95% CI, 8.2-9.2; *P*<.001). **Discussion:** Among nationally insured individuals within the recommended age range for CRC screening, overall adherence to the mt-sDNA test was in the 70s, with the highest rates in the digital (SMS and email) outreach group and the lowest in the no-digital-outreach group. **Conclusions:** These findings highlight the importance of multichannel navigation in facilitating completion of CRC screening with the mt-sDNA test.

## BACKGROUND

Colorectal cancer (CRC) is the fourth most frequently diagnosed cancer and the second leading cause of cancer-related deaths in the United States, accounting for an estimated 152 810 incident cases and 53 010 deaths in 2024.^1,2^ Screening can reduce CRC-related mortality by identifying patients with premalignant or malignant cancers at earlier stages, when treatment and intervention may be more effective.^3^

The US Preventive Services Task Force recommends screening beginning at age 45 for individuals at average risk of developing CRC who have no personal history of CRC, certain polyps, inflammatory bowel disease, abdominal radiation therapy to treat prior cancer, familial history of CRC, or confirmed or suspected hereditary CRC syndrome.^1,4^ Established screening methods for CRC comprise endoscopic evaluation (eg, colonoscopy or sigmoidoscopy), radiologic imaging, and stool-based options, including the high-sensitivity guaiac fecal occult blood test (FOBT), the fecal immunochemical test (FIT), and the multi-target stool DNA (mt-sDNA) test (Cologuard®; Exact Sciences, Madison, Wisc.).^1,5^ Barriers that hinder individuals from utilizing and adhering to some of these established screening techniques may include lack of awareness, concern over test invasiveness, and scheduling challenges.^6^ The mt-sDNA test is among the noninvasive stool-based CRC screening tools recommended by the American Cancer Society.^7^ Unlike conventional invasive screening methods, noninvasive stool-based tests can be performed at home. The added convenience and the high sensitivity of the mt-sDNA test at detecting CRC and advanced precancerous lesions may help improve CRC screening and detection rates.^8,9^

Patient access to screening is a key factor in reducing both the health and financial burden associated with CRC.^3,10^ Additionally, adherence to screening recommendations is crucial in ensuring the effectiveness of screening interventions.^11^ However, according to the National Health Interview Survey, the average up-to-date CRC screening prevalence in the United States for individuals at least 45 years of age was estimated to be only 59% in 2021.^12^ Therefore, there is a need for strategies directed toward increasing CRC screening initiation, as well as the completion of initial and subsequent tests.

As adherence to cancer screening interventions may improve their effectiveness in reducing CRC-specific mortality, strategies with the potential to increase adherence may be an important factor in reducing the treatment burden on patients and the economic impact at both individual and societal levels.^11^ Several modalities and approaches to improve adherence to cancer screening exist, including digital outreach through email and SMS.^13^ Digital outreach via mobile apps, SMS, and wearable devices has been associated with increased treatment adherence to medications in general, as well as increased adherence to screening for cervical cancer.^14,15^ With respect to CRC, prior studies assessing the impact of digital outreach methods on screening uptake with FOBT or FIT found that neither text messages nor email reminders increased adherence, while recent evidence suggests that adherence for FIT may be higher when patients are sent reminders by phone call (20%) compared to text messages (17.7%).^16,17^ At present, research evaluating digital outreach for the mt-sDNA test is scarce. With the mt-sDNA test kits, patients receive access to an enhanced navigation and support system that includes automated and live phone calls, mailed letters, emails, and SMS, which aims to maximize screening adherence. However, the impact of different digital outreach channels on adherence to the mt-sDNA test has not been assessed.

Given the importance of screening adherence in reducing disease burden and insufficient research assessing the impact of digital outreach on CRC screening uptake, evidence is needed to help inform strategies that may increase screening uptake and adherence. Therefore, this study aimed to assess the impact of different digital outreach channels, including SMS plus email, on patient adherence to CRC screening with the mt-sDNA test in a real-world setting.

## METHODS

### Data Source

This retrospective population-based cohort study used laboratory data from Exact Sciences Laboratories, LLC (ESL) from January 1, 2023, to September 23, 2023, to ensure all mt-sDNA orders reach 365 days since shipment. Data were analyzed on September 23, 2024. All data were de-identified and compliant with HIPAA (Health Insurance Portability and Accountability Act).

### Study Design and Sample Selection

Individuals aged 45 to 85 years with commercial, Medicare, Medicaid, and Medicare Advantage insurance who were new to mt-sDNA testing and had a valid order of a collection kit placed by a healthcare provider at the point of care that was shipped between January 1, 2023, and September 23, 2023, were included in this study. The returned test kits containing a specimen was processed and validated by ESL. Patients were excluded if they were less than 45 years of age or more than 85 years of age, had an invalid nonmobile or landline phone number, received a test from any order known to be placed in bulk through a mail-based care gap closure program, or had unknown data fields for sex, payer, and patient median household income by zip code, participant of a ESL-sponsored prospective study, and other irrelevant fields.

### Measures and Outcomes

Patient demographic characteristics included age category, sex, race, ethnicity, geography, payer type, patient median household income by zip code, provider specialty, and preferred language. Patients were given the opportunity to opt out of any of the digital outreach channels via the patient navigation program and were assigned to outreach groups based on their preferences. All patients received standard outreach that included welcomes, reminders (approximately 2-3 times per week for the first 3 weeks, with additional reminders scheduled if test kit was not returned), and notification of test result availability via letters and phone calls on their landline. Participants received additional communication via SMS, email, or both SMS plus email depending on whether there was a mobile phone number and/or email address provided on the order form. Depending on which types of overall communications the participant received on top of the standard outreach, they were grouped into the following 4 outreach groups as follows:

- The fully digital SMS plus email group received both additional SMS plus email reminders.
- The digital SMS group received additional SMS reminders only.
- The digital email group received additional email reminders only.
- The no-digital-communication group received only the standard outreach with no additional SMS texts or emails.

Outcomes included adherence and time to test return. Adherence was defined as the percentage of eligible participants successfully returning a test kit with a specimen within 365 days of initial shipment date (ie, participants returning the test kits outside the 365-day window were considered nonadherent). The time to test return was defined as the number of days from the date of kit shipment to the patient (start date) to the date of receipt of a test kit that contained a specimen by ESL (end date). The time to test return did not include the time for laboratory processing of received tests.

### Statistical Analyses

Categorical variables were summarized using counts and percentages and continuous outcomes were summarized using means. Adherence and the mean number of days to screening were compared across categories of digital outreach (digital SMS plus email, digital SMS, digital email, or no digital communication) stratified by age category, sex, race, ethnicity, geography, payer type, patient income status by zip code, provider specialty, and preferred language. Statistical comparisons to calculate *P* values were conducted using the $\chi^{2}$ test for adherence rates and analysis of variance (ANOVA) for mean days to test return.

Logistic regression analysis was used to evaluate the association of digital outreach method with adherence (binary outcome, yes/no; reference, no digital). Linear regression analysis was used to evaluate time to test return in days (continuous outcome). Covariates were included in the multivariable models if they had significant *P* values in the descriptive, univariable analysis (race, ethnicity, and preferred language were excluded due to missing or incomplete data for most patients). In addition to digital outreach, the following covariates were evaluated for inclusion in both models: age category (reference: 45-49 years), sex (reference: female), geography (reference: metropolitan), payer type (reference: commercial), provider specialty (reference: primary care physician), and patient median household income by zip code (reference: <$50 000). Final regression results were obtained from a single multivariable logistic regression model adjusted for all above-mentioned covariates. For linear regression, the outcome was log-transformed because time to screening was not normally distributed. Statistical analyses were conducted using R Statistical Software version 4.2.2 (R Foundation for Statistical Computing, Vienna, Austria).

## RESULTS

### Patient Characteristics

**Figure 1.**
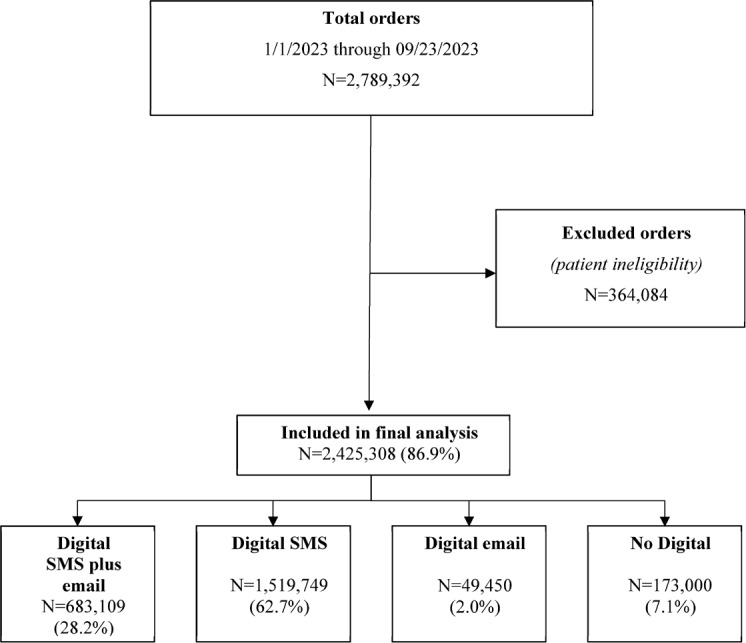
Sample Selection

Among 2 789 392 patients who were shipped mt-sDNA test kits, 2 425 308 (86.9%) met the eligibility criteria and were included in the final analysis (**Figure 1**). Of those included, and based on outreach channels, 28.2% received digital SMS plus email, 62.7% received digital SMS only, 2.0% received digital email only, and 7.1% received no digital outreach. Most patients were female (58.2%) and between 50 and 75 years of age (71.3%) (**Table 1**). Because recording of race, ethnicity, and preferred language are not required on the mt-sDNA order form, more than 64.6% of patients had missing information for these variables. Of those for whom these data were available, substantial proportions of patients were White (21.3%), not of Hispanic or Latino origin or descent (28.5%), and reported English as their preferred language (32.3%). Most patients resided in a metropolitan area (81.6%), resided in a zip code with a median household income between $50 000 and $100 000 (65.7%), and were covered under commercial health insurance (63.9%). Patients in the digital SMS-plus-email group tended to be younger than 65 years and were more likely to select English as their preferred language and have commercial insurance coverage than those in the no-digital-outreach group.

**Table 1.** Patient Characteristics

	**Overall** **(N = 2 425 308), n (%)**	**No Digital** **(N = 173 000), n (%)**	**Digital Email (N = 49 450), n (%)**	**Digital SMS (N = 1 519 749), n (%)**	**Digital Email Plus SMS** **(N = 683 109), n (%)**	***P* Value**
Age category (y)						
45-49	565 275 (23.3)	15 321 (8.9)	5711 (11.5)	357 692 (23.5)	186 551 (27.3)	<.001
50-64	1 054 314 (43.5)	53 943 (31.2)	16 606 (33.6)	673 594 (44.3)	310 171 (45.4)	
65-75	673 884 (27.8)	76 427 (44.2)	19 628 (39.7)	418 677 (27.5)	159 152 (23.3)	
76-85	131 835 (5.4)	27 309 (15.8)	7 505 (15.2)	69 786 (4.6)	27 235 (4.0)	
Sex						
Female	1 412 369 (58.2)	101 390 (58.6)	30 541 (61.8)	871 308 (57.3)	409 130 (59.9)	<.001
Male	1 012 939 (41.8)	71 610 (41.4)	18 909 (38.2)	648 441 (42.7)	273 979 (40.1)	
Race						
White	517 006 (21.3)	37 005 (21.4)	11 029 (22.3)	301 779 (19.9)	167 193 (24.5)	<.001
Black or African American	56 068 (2.3)	3 633 (2.1)	842 (1.7)	35 212 (2.3)	16 381 (2.4)	
American Indian or Alaska Native	2748 (0.1)	152 (0.1)	60 (0.1)	1689 (0.1)	847 (0.1)	
Asian or Indian	24 995 (1.0)	920 (0.5)	473 (1.0)	14 951 (1.0)	8651 (1.3)	
Other	2323 (0.1)	102 (0.1)	15 (0.0)	1572 (0.1)	634 (0.1)	
Unknown	1 822 168 (75.1)	131 188 (75.8)	37 031 (74.9)	1 164 546 (76.6)	489 403 (71.6)	
Ethnicity						
Not of Hispanic or Latino origin or descent	690 281 (28.5)	49 857 (28.8)	11 432 (23.1)	438 217 (28.8)	190 775 (27.9)	<.001
Hispanic or Latino origin or descent	85 569 (3.5)	3417 (2.0)	611 (1.2)	60 213 (4.0)	21 328 (3.1)	
Other	1008 (0.0)	35 (0.0)	4 (0.0)	727 (0.0)	242 (0.0)	
Unknown	1 648 450 (68.0)	119 691 (69.2)	37 403 (75.6)	1 020 592 (67.2)	470 764 (68.9)	
Geography						
Metropolitan	1 978 370 (81.6)	129 631 (74.9)	39 858 (80.6)	1 232 410 (81.1)	576 471 (84.4)	<.001
Micropolitan	245 671 (10.1)	21 055 (12.2)	4 943 (10.0)	158 149 (10.4)	61 524 (9.0)	
Rural	78 722 (3.2)	10 142 (5.9)	2 100 (4.2)	49 314 (3.2)	17 166 (2.5)	
Small town	122 545 (5.1)	12 172 (7.0)	2549 (5.2)	79 876 (5.3)	27 948 (4.1)	
Payer type						
Commercial	1 550 150 (63.9)	68 573 (39.6)	23 710 (47.9)	975 277 (64.2)	482 590 (70.6)	<.001
Medicare Advantage	501 465 (20.7)	55 362 (32.0)	12 477 (25.2)	319 952 (21.1)	113 674 (16.6)	
Medicaid	40 848 (1.7)	2437 (1.4)	430 (0.9)	28 922 (1.9)	9059 (1.3)	
Medicare	332 845 (13.7)	46 628 (27.0)	12 833 (26.0)	195 598 (12.9)	77 786 (11.4)	
Median household income by patient zip code						
<$50 000	262 507 (10.8)	22 544 (13.0)	4 637 (9.4)	173 536 (11.4)	61 790 (9.0)	<.001
50 000−75 000	958 658 (39.5)	71 678 (41.4)	17 899 (36.2)	613 638 (40.4)	255 443 (37.4)	
75 000−100 000	635 544 (26.2)	41 596 (24.0)	12 507 (25.3)	395 532 (26.0)	185 909 (27.2)	
100 000−125 000	342 006 (14.1)	21 687 (12.5)	7634 (15.4)	205 756 (13.5)	106 929 (15.7)	
>$125 000	226 593 (9.3)	15 495 (9.0)	6773 (13.7)	131 287 (8.6)	73 038 (10.7)	
Preferred language						
English	783 524 (32.3)	38 905 (22.5)	22 496 (45.5)	421 015 (27.7)	301 108 (44.1)	<.001
Spanish	62 581 (2.6)	1856 (1.1)	303 (0.6)	42 587 (2.8)	17 835 (2.6)	
Other	12 573 (0.5)	456 (0.3)	114 (0.2)	8790 (0.6)	3213 (0.5)	
Unknown	1 566 630 (64.6)	131 783 (76.2)	26 537 (53.7)	1 047 357 (68.9)	360 953 (52.8)	
Provider specialty						
Primary care physician	1 495 720 (61.7)	111 098 (64.2)	31 692 (64.1)	939 867 (61.8)	413 063 (60.5)	<.001
Gastroenterologist	46 256 (1.9)	6193 (3.6)	1586 (3.2)	26 126 (1.7)	12 351 (1.8)	
Nurse practitioner/ physician assistant	680 233 (28.0)	43 401 (25.1)	11 853 (24.0)	432 965 (28.5)	192 014 (28.1)	
Obstetrician/ gynecologist	84 025 (3.5)	3613 (2.1)	2019 (4.1)	46 632 (3.1)	31 761 (4.6)	
Other	117 686 (4.9)	8618 (5.0)	2269 (4.6)	73 289 (4.8)	33 510 (4.9)	
Unknown	1388 (0.1)	77 (0.0)	31 (0.1)	870 (0.1)	410 (0.1)	

### Adherence and Mean Time to Test Return

For test adherence, the overall nonempty return rate of mt-sDNA test within 365 days of kit receipt was 70.1% (**Table 2**), with the highest adherence in the digital SMS-plus-email group (72.9%) and lowest adherence in the no-digital group (63.4%). Adherence was highest in the digital SMS-plus-email group across nearly all patient characteristics (*P* < .001 for almost all categories), except for the relatively smaller category of “other ethnicity.” Adherence was numerically highest in the oldest age group (76.3%), with similar levels of adherence in females (70.1%) and males (70.0%); these observations were consistent across categories of digital outreach. Adherence increased with zip code median household income and varied within payer type, with numerically highest adherence observed in patients insured through Medicare payers (79.4%) and lowest adherence in those covered through Medicaid (48.3%). Additionally, adherence was numerically similar (~70%) among patients residing in metropolitan, micropolitan, rural, or small-town areas. Among healthcare providers, mt-sDNA tests prescribed by gastroenterologists achieved the highest adherence rate (80.4%) compared with other provider specialties.

**Table 2.** Adherence Rates^a^ by Type of Digital Outreach, Overall and by Patient Characteristics

Adherence,^a^ %						
	**Overall** **(N = 2 425 308)**	**No Digital (N = 173 000)**	**Digital Email (N = 49 450)**	**Digital SMS (N = 1 519 749)**	**Digital Email Plus SMS** **(N = 683 109)**	***P* Value**
Overall	70.1	63.4	68.5	69.6	72.9	<.001
Age category (y)						
45-49	69.0	58.7	62.2	68.7	70.8	<.001
50-64	68.5	58.4	64.1	68.0	71.5	<.001
65-75	72.2	65.0	70.9	71.8	76.6	<.001
76-85	76.3	71.4	76.7	76.3	80.9	<.001
Sex						
Female	70.1	63.5	67.6	69.7	72.6	<.001
Male	70.0	63.2	69.9	69.4	73.3	<.001
Race						
White	66.9	61.0	62.3	66.8	68.7	<.001
Black or African American	58.4	52.6	52.0	57.9	61.1	<.001
American Indian or Alaska Native	55.6	51.3	48.3	55.0	58.0	.205
Asian or Indian	68.7	63.9	59.4	69.0	69.3	<.001
Other	54.7	42.2	53.3	53.9	58.7	.012
Unknown	71.4	64.4	70.9	70.7	74.8	<.001
Ethnicity						
Not of Hispanic or Latino origin or descent	69.2	62.4	63.0	69.1	71.4	<.001
Hispanic or Latino origin or descent	64.1	60.1	58.6	64.1	65.0	<.001
Other	64.5	60.0	25.0	63.1	69.8	.078
Unknown	70.7	63.9	70.3	70.1	73.8	<.001
Geography						
Metropolitan	70.0	63.0	68.1	69.5	72.6	<.001
Micropolitan	70.7	64.8	70.6	70.1	74.3	<.001
Rural	70.4	64.3	68.7	70.3	74.5	<.001
Small town	69.9	64.4	69.9	69.3	73.7	<.001
Payer type						
Commercial	69.0	55.5	60.3	69.0	71.4	<.001
Medicare Advantage	68.8	64.6	70.5	68.0	73.0	<.001
Medicaid	48.3	41.2	49.3	47.3	53.3	<.001
Medicare	79.4	74.8	82.3	78.5	83.9	<.001
Median household income by patient zip code						
<$50 000	64.2	58.7	65.8	63.4	68.4	<.001
50 000−75 000	69.1	63.6	68.1	68.6	71.8	<.001
75 000−100 000	71.3	64.6	68.4	71.0	73.7	<.001
100 000−125 000	72.5	64.3	69.5	72.4	74.6	<.001
>$125 000	73.6	64.9	70.4	73.6	75.8	<.001
Preferred language						
English	68.9	60.1	66.5	67.8	71.8	<.001
Spanish	67.7	63.8	61.7	67.7	68.1	<.001
Other	71.3	60.5	70.2	71.2	73.2	<.001
Unknown	70.7	64.4	70.3	70.4	74.0	<.001
Provider specialty						
Primary care physician	71.1	64.1	69.1	70.7	74.0	<.001
Gastroenterologist	80.4	77.7	81.4	79.8	82.9	<.001
Nurse practitioner/ physician assistant	67.0	59.7	65.4	66.5	70.0	<.001
Obstetrician/gynecologist	71.2	62.7	67.1	71.3	72.5	<.001
Other	69.1	62.1	68.0	68.3	72.7	<.001
Unknown	69.3	63.6	41.9	67.9	75.4	<.001

^a^Defined as the percentage of eligible participants successfully returning a nonempty test kit within 365 days of initial shipment date.

The mean overall time to test return (days) from shipment of mt-sDNA kit to receipt of a valid nonempty test was 25.8 days (**Table 3**). Across the categories of digital outreach, the time to test return ranged from 25.1 days in the digital SMS-plus-email group to 28.7 days in the no-digital group.

**Table 3.** Time to Test Return by Type of Digital Outreach, Overall and by Patient Characteristics

**Time to Test Return,^a^ Mean Days (SD)**						
	**Overall** **(N = 2 425 308)**	**No Digital (N = 173 000)**	**Digital Email (N = 49 450)**	**Digital SMS (N = 1 519 749)**	**Digital Email Plus SMS** **(N = 683 109)**	***P* Value**
Overall	25.8 (40.5)	28.7 (45.9)	27.1 (43.1)	25.9 (40.3)	25.1 (39.5)	<.001
Age category (y)						
45-49	28.6 (45.0)	40.3 (60.4)	35.8 (55.4)	28.7 (44.9)	27.6 (43.5)	<.001
50-64	26.8 (41.5)	33.9 (51.9)	31.4 (47.4)	26.7 (41.1)	25.8 (40.3)	<.001
65-75	23.4 (36.5)	26.2 (41.9)	24.4 (39.0)	23.3 (36.1)	22.1 (34.6)	<.001
76-85	20.1 (31.0)	21.1 (33.9)	20.3 (32.6)	20.2 (30.3)	19.2 (29.5)	<.001
Sex						
Female	26.6 (41.5)	29.1 (45.9)	28.2 (44.9)	26.6 (41.3)	25.8 (40.5)	<.001
Male	24.8 (39.1)	28.1 (45.7)	25.4 (40.1)	24.9 (38.8)	24.0 (38.0)	<.001
Race						
White	25.9 (41.0)	28.1 (45.8)	28.1 (44.9)	25.7 (40.5)	25.6 (40.8)	<.001
Black or African American	25.3 (40.0)	26.1 (41.2)	25.9 (41.3)	25.4 (40.0)	24.9 (39.7)	.564
American Indian or Alaska Native	29.4 (46.1)	30.8 (37.7)	23.7 (29.4)	30.0 (48.1)	28.4 (44.4)	0.83
Asian or Indian	24.7 (39.7)	29.9 (50.1)	27.4 (44.1)	24.6 (39.6)	24.3 (38.5)	.006
Other	26.0 (39.4)	30.1 (40.9)	12.8 (5.3)	27.2 (41.8)	23.1 (33.6)	.244
Unknown	25.9 (40.4)	28.9 (45.9)	26.9 (42.7)	25.9 (40.3)	25.0 (39.2)	<.001
Ethnicity						
Not of Hispanic or Latino origin or descent	25.8 (41.0)	28.5 (45.9)	26.5 (43.4)	25.7 (40.5)	25.2 (40.7)	<.001
Hispanic or Latino origin or descent	25.9 (38.8)	27.8 (40.4)	28.0 (41.5)	26.0 (38.9)	25.5 (38.2)	.051
Other	27.1 (40.8)	45.7 (73.3)	9.0 (NA)	26.3 (35.3)	27.3 (48.4)	.19
Unknown	25.9 (40.4)	28.8 (46.0)	27.3 (43.1)	25.9 (40.3)	25.0 (39.1)	<.001
Geography						
Metropolitan	26.0 (41.1)	29.1 (46.8)	27.3 (43.7)	26.1 (40.9)	25.2 (40.0)	<.001
Micropolitan	24.9 (37.9)	27.2 (42.7)	26.0 (40.2)	24.9 (37.6)	24.2 (37.0)	<.001
Rural	26.1 (38.9)	28.3 (43.6)	26.2 (36.6)	26.0 (38.9)	25.3 (36.5)	<.001
Small town	24.9 (37.7)	27.3 (43.2)	27.5 (44.5)	24.6 (36.5)	24.6 (37.9)	<.001
Payer type						
Commercial	27.1 (42.4)	34.4 (52.5)	31.8 (49.0)	27.1 (42.1)	26.2 (41.2)	<.001
Medicare Advantage	23.7 (36.5)	25.2 (40.2)	23.8 (37.2)	23.8 (36.4)	22.7 (35.0)	<.001
Medicaid	25.7 (37.9)	29.1 (43.0)	30.2 (47.5)	25.6 (37.9)	25.0 (36.4)	.005
Medicare	23.3 (37.7)	26.0 (42.9)	23.5 (38.2)	23.2 (37.2)	22.1 (35.9)	<.001
Median household income by patient zip code						
<$50 000	24.9 (37.4)	26.7 (42.2)	25.5 (37.5)	24.9 (37.2)	24.3 (36.5)	<.001
50 000−75 000	25.4 (39.1)	28.0 (44.5)	26.6 (41.6)	25.3 (38.7)	24.7 (38.2)	<.001
75 000−100 000	26.1 (41.1)	29.5 (46.8)	27.3 (43.0)	26.2 (41.1)	25.2 (39.8)	<.001
100 000−125 000	26.6 (42.7)	30.6 (49.8)	28.0 (46.4)	26.7 (42.8)	25.5 (40.9)	<.001
>$125 000	26.9 (44.1)	29.5 (47.9)	28.3 (46.6)	26.9 (44.0)	26.2 (43.2)	<.001
Preferred language						
English	26.1 (41.3)	28.2 (45.5)	27.3 (43.3)	26.3 (41.5)	25.4 (40.4)	<.001
Spanish	25.8 (36.7)	27.0 (38.9)	30.8 (52.0)	25.7 (36.4)	25.9 (36.8)	.158
Other	22.3 (34.2)	21.3 (26.3)	24.6 (49.3)	22.8 (35.1)	20.8 (32.0)	.092
Unknown	25.8 (40.3)	28.9 (46.1)	27.0 (42.9)	25.7 (40.0)	24.8 (39.0)	<.001
Provider specialty						
Primary care physician	25.9 (40.9)	28.8 (46.1)	26.7 (42.5)	25.9 (40.7)	25.1 (39.9)	<.001
Gastroenterologist	21.6 (35.1)	23.3 (39.5)	21.6 (35.8)	21.4 (33.9)	21.1 (35.0)	.003
Nurse practitioner/ physician assistant	25.9 (39.5)	29.0 (45.6)	28.6 (45.5)	25.9 (39.1)	25.2 (38.6)	<.001
Obstetrician/gynecologist	27.9 (44.7)	33.1 (51.7)	30.4 (46.6)	28.2 (45.2)	26.9 (43.0)	<.001
Other	25.8 (40.0)	28.8 (46.1)	27.4 (42.6)	25.9 (39.9)	24.9 (38.6)	<.001
Unknown	25.5 (36.5)	28.0 (39.4)	18.8 (35.4)	25.7 (36.9)	25.0 (35.4)	.861

^a^Defined as the number of days from date of kit shipment to patient to the date of receipt of a nonempty test by ESL (time to screening does not include time for lab processing of received tests).

### Summary of Model Output

Logistic regression analysis showed that patients in the digital SMS-plus-email group had the highest odds of returning their test (odds ratio [OR], 1.75; 95% confidence interval [CI], 1.73-1.78); those receiving SMS only and those receiving email only had higher odds of returning their tests compared with the no-digital-outreach group (**Table 4**; all *P* < .001). Patients aged 76 to 85 years had higher odds of adherence compared with 45 to 49-year-olds (OR, 1.24; 95% CI, 1.22-1.26; *P* < .001).

**Table 4.** Multivariable Regression Results for Association of Outreach Type With Adherence and Time to Test Return

	**Adherence Rate: Logistic Regression**			**Log-Transformed Days to Test Return: Linear Regression**		
	**Odds Ratio^a^**	**95% CI**	***P* Value**	**Estimate^b^**	**95% CI**	***P* Value**
Intercept	1.12	(1.10, 1.14)		19.90	(19.771, 20.038)	
Outreach type						
No digital	Ref			Ref		
Digital SMS plus email	1.75	(1.73, 1.77)	<.001	0.91	(0.90, 0.91)	<.001
Digital email only	1.24	(1.22, 1.27)	<.001	0.95	(0.94, 0.96)	<.001
Digital SMS only	1.49	(1.48, 1.51)	<.001	0.95	(0.94, 0.95)	<.001
Age category (y)						
45-49	Ref			Ref		
50-64	1.00	(0.99, 1.00)	.937	0.95	(0.95, 0.95)	<.001
65-75	1.04	(1.03, 1.05)	<.001	0.85	(0.85, 0.86)	<.001
76-85	1.23	(1.21, 1.25)	<.001	0.76	(0.76, 0.77)	<.001
Sex						
Female	Ref			Ref		
Male	1.00	(0.99, 1.00)	.73	0.94	(0.93, 0.94)	<.001
Geography						
Metropolitan	Ref			Ref		
Micropolitan	1.16	(1.15, 1.17)	<.001	1.00	(1.00, 1.01)	.015
Rural	1.19	(1.17, 1.21)	<.001	1.07	(1.06, 1.08)	<.001
Small town	1.15	(1.13, 1.16)	<.001	1.01	(1.01, 1.02)	<.001
Payer type						
Commercial	Ref			Ref		
Medicare Advantage	1.01	(1.00, 1.02)	.005	0.98	(0.98, 0.99)	<.001
Medicaid	0.45	(0.44, 0.45)	<.001	0.97	(0.96, 0.98)	<.001
Medicare	1.70	(1.68, 1.72)	<.001	0.97	(0.97, 0.97)	<.001
Median household income by patient zip code						
<$50 000	Ref			Ref		
50 000−75 000	1.23	(1.21, 1.24)	<.001	1.00	(1.00, 1.00)	.002
75 000−100 000	1.38	(1.37, 1.40)	<.001	1.01	(1.01, 1.02)	<.001
100 000−125 000	1.46	(1.45, 1.48)	<.001	1.01	(1.01, 1.02)	<.001
>$125 000	1.52	(1.50, 1.54)	<.001	1.00	(0.99, 1.00)	.719
Provider specialty						
Primary care physician	Ref			Ref		
Gastroenterologist	1.52	(1.48, 1.55)	<.001	0.88	(0.88, 0.89)	<.001
Nurse practitioner/ physician assistant	0.85	(0.85, 0.86)	<.001	1.01	(1.00, 1.01)	<.001
Obstetrician/gynecologist	0.93	(0.83, 1.04)	.255	1.00	(0.95, 1.05)	.837
Other	1.02	(1.00, 1.04)	<.001	1.00	(0.99, 1.01)	.083
Unknown	0.91	(0.90, 0.92)	<.001	1.00	(1.00, 1.01)	.002

^a^Odds ratio is defined as the odds of adherence in a particular group relative to the odds of adherence in the Ref group.

^b^These estimates are exponentiating the coefficients and their CIs to get the multiplicative effect of each on time to return.

Compared with individuals living in metropolitan areas, those living in micropolitan areas (OR, 1.16; 95% CI, 1.15-1.17), rural areas (OR, 1.19; 95% CI, 1.17-1.21), and small towns (OR, 1.15; 95% CI, 1.13-1.16) had higher odds of adherence (all *P* < .001). Relative to patients with commercial insurance, those with Medicare coverage had higher odds of adherence (OR, 1.70; 95% CI, 1.67-1.72, *P* < .001). Patients who were followed by a gastroenterologist had 52% higher odds of adherence than those followed by a primary care physician (OR, 1.52; 95% CI, 1.49-1.56; *P* < .001). Relative to patients who lived in a zip code with median household income less than $50 000, all patients living in zip codes with higher median household incomes had higher odds of returning valid nonempty mt-sDNA kits within 365 days from shipment (all *P* < .001).

Log-linear regression analysis showed that compared with the no-digital-outreach group, patients who received any type of digital outreach had relatively faster return times: digital SMS-plus-email was 8.7% faster in return kit order (95% CI, 8.2-9.2), digital email-only was 4.8% faster (95% CI, 3.9-5.7), and digital-SMS-only was 4.8% faster (95% CI, 4.3-5.3) (*P* < .001 for all).

## DISCUSSION

In this large study population of nationally insured individuals encompassing the age ranges recommended for average-risk CRC screening, the adherence rate for the mt-sDNA test was 70.1% overall, with numerically highest rates in the digital SMS-plus-email outreach group (72.9%) and lowest rates in the no-digital-outreach group (63.4%). Although differences in time to test return across categories of digital outreach were statistically significant, the absolute differences were moderate, ranging from 25.1 days in the digital SMS-plus-email outreach group to 28.7 days in the no-digital-outreach group. After adjustment for potential confounding factors, such as age, sex, and zip code household income, patients who received digital SMS plus email outreach had a higher likelihood of adherence than patients with no digital outreach.

Literature evaluating the effect of digital outreach on CRC screening adherence is scarce.^13,16^ In a randomized trial assessing the effect of SMS reminders on FOBT adherence, no difference in return rates was found when compared with patients who received no text message reminder.^18^ In another randomized trial that assessed different outreach modalities for FIT adherence, it was found that patients receiving SMS alone had lower adherence rates (17%) than those receiving letter mail (24%), while live phone calls or live phone calls combined with SMS had adherence rates of 32% and 27%, respectively.^19^ Similarly, the results of a recent stepped-wedge trial indicated that those receiving reminders through live phone calls had higher FIT adherence (20%) than those receiving text messages alone (18%).^17^ While these adherence outcomes across screening modalities could not be directly compared because of differences in study design and population, relative to FOBT and FIT adherence rates observed in the literature, adherence to mt-sDNA screening in this study appeared higher overall (65%), and adherence rates were highest when both SMS plus email were used. These findings may indicate that the outreach strategies within the patient navigation system for the mt-sDNA test may be more effective at increasing screening adherence in the studied population than those used in the FOBT/FIT studies, where phone calls and/or text messages were used exclusively. This study contributes to our understanding of the effect of digital outreach on adherence to CRC screening in a real-world setting and provides some evidence that use of both SMS plus email may increase adherence to the mt-sDNA test compared with other forms of outreach.

Patients in the present study had the option to opt out of specific digital outreach channels; therefore, individual choices may reflect a patient’s willingness to adhere. Nonetheless, adherence to mt-sDNA test for those receiving digital SMS and email outreach (72.9%) was higher than the overall adherence reported for the same test in a study of a Medicaid-insured population (51%), and similar to that observed in a study of a nationally insured population (67%).20,21 Meanwhile, the outreach group with the lowest adherence in this study was still nearly 63.4% adherent. These results reflect high adherence across all categories of outreach and suggest that the flexible strategies for outreach offered through the mt-sDNA patient navigation system may be well suited to improving screening adherence, while meeting the diverse preferences of screening patients.

The use of digital outreach modalities, such as SMS plus email, appears to be a simple and effective way to improve adherence to CRC screening with mt-sDNA tests. This may help reduce patient burden and mortality by detecting CRC earlier, when it is more easily treated. While the national average CRC screening rate of 59% remains to be improved overall,^12^ the results of this study also identified specific populations that may benefit from strategies that aim to improve adherence, such as patients in lower zip code household income categories, who tended to have worse adherence than those with higher zip code household income. Lower socioeconomic status and income have been found to be associated with higher incidence and mortality of CRC, with financial strain being a potential risk associated with reduced CRC screening.^20^ Therefore, strategies to improve access to CRC screening in those of lower socioeconomic status could be considered to reduce CRC mortality in this vulnerable group. In addition, given that younger age groups have only recently been included in CRC screening recommendations, awareness may be lacking in both patients and healthcare providers alike, and thus more effort is required to encourage these age groups to complete screening.^4^

Overall, the results of this study are encouraging, as any strategy that increases screening adherence may increase the chance of detecting CRC at earlier stages when the tumor is easier to treat,^3^ which could potentially improve quality of life among patients and may reduce the societal and individual burden associated with intensive medical treatment, as well as the cumulative personal/familial financial and medical costs associated with CRC in the long run.^21–23^ Future prospective study randomizing mt-sDNA users to different outreach strategies may help establish causal relationships between different strategies and adherence. Further research is also warranted, focusing on specific groups with lower adherence, such as patients using different payer types or those in lower-income households.

### Limitations

The findings of the present analysis are subject to certain limitations. Notably, there was a high amount of missing data for race, ethnicity, and preferred language information. Meanwhile, certain demographic groups (eg, metropolitan residents, individuals with commercial insurance) were more highly represented, which may affect the generalizability of the results. Although some patient characteristics were adjusted for in the multivariable regression models, residual or unmeasured confounding cannot be ruled out. Additionally, data were limited to what was available from ESL laboratory data; as such, the number of CRC cases identified following a positive mt-sDNA test could not be assessed. Future studies may also access the potential impact of physician involvement on adherence to mt-sDNA test and timing of follow up testing. Lastly, as patients in this study were assigned to a digital outreach group according to which digital channels they opted out of, this self-selection may reflect an increased willingness to adhere to testing and should be considered when interpreting the finding.

## CONCLUSIONS

This large national study of insured patients with a valid order for CRC screening with the mt-sDNA test demonstrated that, of all the digital outreach methods assessed, those who received both SMS plus email had the highest rate of adherence to the mt-sDNA screening test. Taken together, the results of this study underscore the importance of multichannel navigation in facilitating the completion of CRC screening with the mt-sDNA test.

### Disclosures

M.G., T.P., A.B.O., and P.L. are employees of Exact Sciences Corporation and own stock/stock options. J.B.K. is an inventor of Mayo Clinic intellectual property under license to Exact Sciences and has received grant support from a sponsored research agreement between Mayo Clinic and Exact Sciences. A.M.F. has been a consultant for AbbVie, Amgen, Centivo, Community Oncology Association, Covered California, EmblemHealth, Exact Sciences, Freedman Health, GRAIL, Harvard University, Health & Wellness Innovations, Health at Scale Technologies, MedZed, Penguin Pay, Risalto, Sempre Health, the State of Minnesota, US Department of Defense, Virginia Center for Health Innovation, Wellth, and Zansors; has received research support from the Agency for Healthcare Research and Quality, Gary and Mary West Health Policy Center, Arnold Ventures, National Pharmaceutical Council, Patient-Centered Outcomes Research Institute, Pharmaceutical Research and Manufacturers of America, the Robert Wood Johnson Foundation, the State of Michigan, and the Centers for Medicare and Medicaid Services.

### Data Availability Statement

The data that support the findings of this study are available from Exact Sciences Laboratories, LLC. Data are available from the authors with the permission of Exact Sciences Laboratories, LLC.

### Ethics Statement

The study was considered exempt research under 45 CFR § 46.104(d)(4) as data were de-identified and compliant with the Health Insurance Portability and Accountability Act (HIPAA), specifically, 45 CFR § 164.514.
